# RNA-seq with RNase H-based ribosomal RNA depletion specifically designed for *C. elegans*

**DOI:** 10.17912/micropub.biology.000312

**Published:** 2020-09-22

**Authors:** Ye Duan, Yongming Sun, Victor Ambros

**Affiliations:** 1 University of Massachusetts Medical School, Worcester, MA 01605, USA; 2 Integrated DNA Technologies, Inc., Redwood City, CA 94065, USA

**Figure 1:**
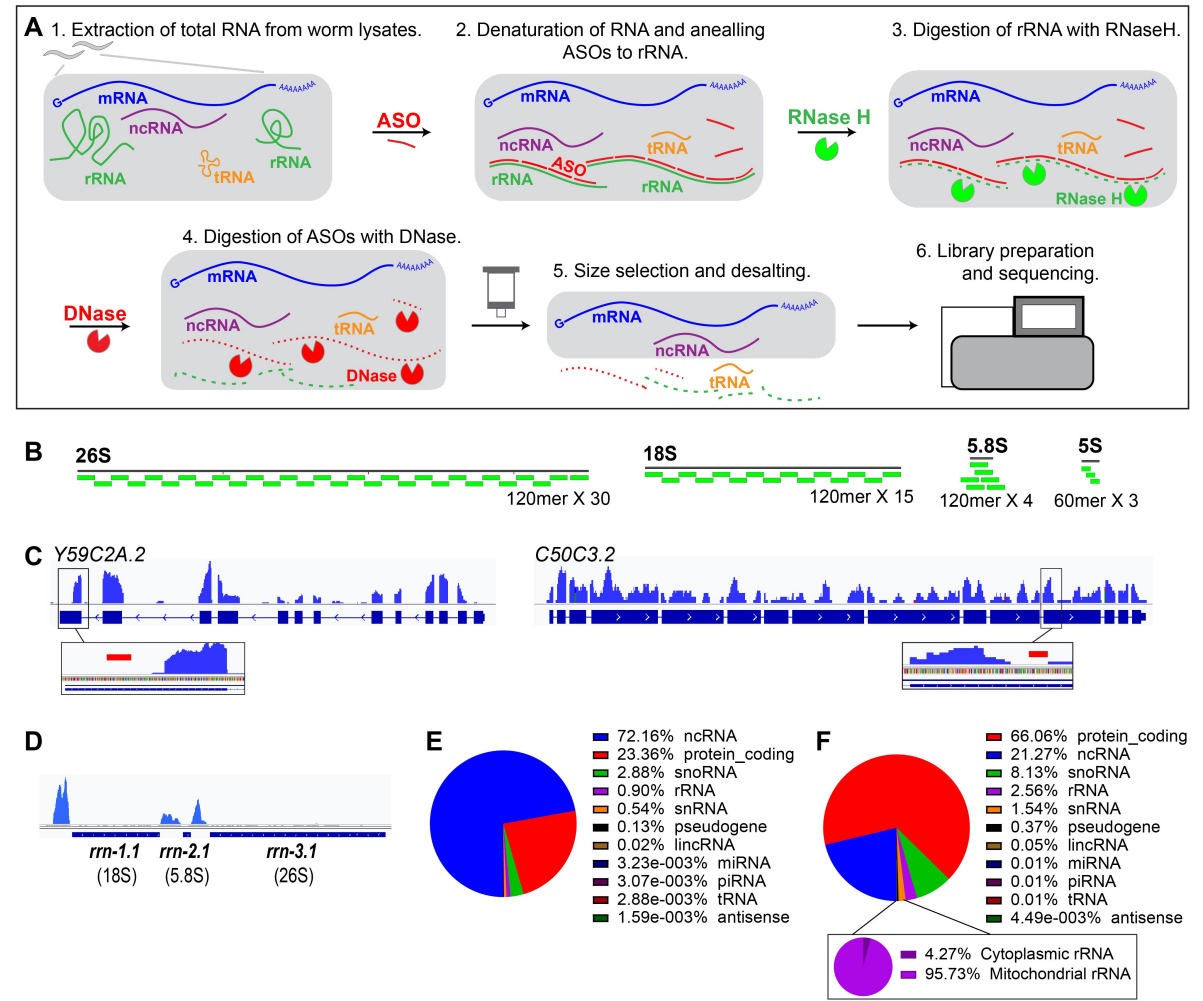
**A.** Protocol for depletion of ribosomal RNAs (rRNA) from samples of *C. elegans* RNA. Total RNA was extracted from worm lysates, heat denatured to melt secondary structure, and hybridized to a molar excess of the DNA antisense oligo (ASO) mixture. rRNA annealed to their complementary oligonucleotides were digested by thermostable RNase H, followed by removal of genomic DNA and ASOs with DNase. The reaction was then passed through a size selection column to desalt the sample and remove the truncated rRNA fragments and small RNAs less than 150 nt (e.g., tRNAs), and the RNA was cloned and sequenced. **B.** Alignments of ASOs to *C. elegans* 26S, 18S, 5.8S and 5Sribosome RNAs (rRNA). Some ASOs have complementarity to both a 5’ segment and a 3’ segment of 5.8S sequence. Images are primarily generated by SnapGene Viewer 5.1.4.1. **C.** Sequencing read coverage (linear scale; image generated by IGV 2.7.2 (Robinson *et al.*, 2011)) at the *Y59C2A.2* and *C50C3.2* mRNA loci, corresponding to the top two predicted off-target sites of ASOs (red bar; see text for discussion). Note that the off-target depletion of mRNA sequence appears to be local and does not appreciably impact read density in other regions of those genes. Reads were from 4 libraries of different genotypes. **D.** Sequencingread coverage (linear scale) at the *C. elegans* primary rDNA locus from 4 libraries of different genotypes. We suggest that the reads flanking the rRNA come from primary rRNA transcripts, which were not targeted by ASOs (Ellis *et al.*, 1986). **E.** Biotype proportions of reads from 12 RNA-seq libraries of 4 different genotypes prepared based on the rRNA depletion. **F.** Biotype proportions of reads in (**E**) after manually removal of signal recognition particle (srpR) RNA reads, which are predominantly enriched. The lower panel shows the proportions of the cytoplasmic rRNA reads (with ASOs) and mitochondrial rRNA reads (without ASOs) among the rRNA reads.

## Description

RNA-seq is widely used for the quantitative analysis of transcriptomes in the context of studies of gene expression and regulation (Mortazavi *et al.*, 2008; Ozsolak and Milos, 2011; Wang *et al.*, 2009). Generally, RNA-seq protocols employ poly(A) selection for mRNA enrichment. However, poly(A) based enrichment is subject to potential bias depending on the poly(A) status of various mRNAs, which could be particularly undesirable in the context of studying post-transcriptional gene regulatory mechanisms, such as miRNA repression (Wu *et al.*, 2006). Therefore, ribosomal RNA (rRNA) depletion is a desirable alternative strategy to enrich for mRNA sequences in RNA-seq sample preparation (Zhao *et al.*, 2014). However, currently available rRNA depletion toolkits were designed for either mammals or bacteria, and hence do not offer an efficient option for rRNA depletion of RNA samples from certain experimental organisms, such as *C. elegans*.

Here we describe a rRNA depletion protocol based on RNase H digestion using antisense oligonucleotides (ASOs) specifically designed for *C. elegans* cytoplasmic rRNA (Fig. 1A). We suggest that this rRNA depletion protocol is applicable to RNA-seq applications where the yield of mRNA enrichment should be independent of poly(A) status, or any application which benefits from the removal of rRNA sequences, such ribosome profiling, or sequencing of non-coding RNAs other than rRNA.

We designed 120mer DNA ASOs complementary to 26S, 18S and 5.8S rRNA sequences and 60mer ASOs complementary to 5S rRNA (Fig. 1B). We used thermostable RNase H and optimized protocols at high temperature for the ASO-rRNA duplex annealing and digestion to reduce the formation of secondary structure of rRNA and off-target annealing of the ASOs. We found that among the 12 RNA-seq libraries (4 different *C. elegans* genotypes) prepared with our rRNA depletion protocol, the cytoplasmic rRNA (26S, 18S, 5.8S and 5S) reads only comprised < 0.2 % of the total reads (Fig. 1E-F), indicating that our protocol is effective for removing rRNA. Highlighting the specificity of the depletion, we observed reads from rRNA precursor transcripts (Fig. 1D) and mitochondrial rRNA (Fig. 1F, lower panel) due to the absence of ASOs against those sequences. As expected, the rRNA-depleted datasets contained a large representation of other non-coding RNA sequences, further supporting that our protocol enriches for RNAs lacking poly(A) (Fig. 1E-F). The performance of our depletion protocol appears to be similar to that of two independently developed similar protocols for *C. elegans*-specific rRNA depletion (Arribere *et al.*, 2016; Barucci *et al.*, 2020). Interestingly, we found that a large proportion of the non-coding RNA reads after RNA depletion correspond to the signal recognition particle RNA (srpR) (Regalia *et al.*, 2002). A similar (although somewhat less prominent) enrichment of srpR was also observed in the libraries of Barucci *et al.*, 2020 and Arribere *et al.*, 2016. The srpR reads were manually removed in our analysis (Fig. 1F); however we suggest that later iterations of our protocol could include ASOs corresponding to srpR, as well as mitochondrial rRNA and cellular primary rRNA transcripts.

Although we did not directly compare the genome-wide distribution of mRNA reads from this ASO protocol to that of mRNA reads from other approaches for mRNA enrichment, we did conduct a computational assessment of the potential for off-target effects of our ASO rRNA depletion. We adopted a conservative assumption that, for an ASO to trigger RNase H digestion of an mRNA sequence, it should match the mRNA with a melting temperature of no less than 20^o^C below the temperature at which the annealing and RNase H digestion were performed. Using BLAST, we identified only two potential matches between the ASOs and the *C. elegans* transcriptome that met this criterion (Morgulis *et al.*, 2008). Interestingly, these two sites do seem to locate in regions of their respective transcriptional units where reads were relatively depleted locally, suggesting that the ASOs may have triggered off-target depletion at these sites (Fig. 1C). However, the apparent sequence depletion in these two instances was only restricted to regions around the ASO off-target match sites, and the alignment distribution for each gene in sequences other than the off-target sites seems to be relatively unaffected (Fig. 1C). From this analysis, we suggest that the ASO rRNA depletion method results in essentially negligible off-target mRNA depletion.

## Methods

**rRNA depletion and RNA-seq**

Harvested worms were washed with M9 medium and flash-frozen in liquid nitrogen. The worm pellets were lysed by QIAzol (Qiagen, Cat: 79306) as previously described (McJunkin and Ambros, 2017). ASOs and total RNA were mixed with approximate 2:1 molar ratio (the quantity of ASOs calculated assuming rRNAs comprise > 90% of total RNA). Higher molar ratios of up to 6.5:1 were tested but did not result in observable improvement in depletion efficacy. Accordingly, a mixture containing final concentrations of 0.1 mM of each ASO, 50 ng/µl total RNA, 10 mM Tris-HCl (pH = 8.0), 100 mM NaCl, 1 mM EDTA and 0.8 U/µl RNase Inhibitor (NEB, Cat:M0314) was incubated at 95 °C for 2 min for denaturation and then 65 °C for 30 min for annealing. We have also tested annealing conditions with gradual decrease in temperature (0.5-2 °C per minute) but no apparent improvement in depletion efficacy was observed. To digest the rRNA, thermostable RNase H and the buffer (MCLAB, Cat:HTRH-100) were preheated to 65 °C and added to the reaction (while maintaining the reaction at 65 °C) to obtain a final concentration of 0.2 U/µl RNase H and the reaction was further incubated at 65 °C for 40 min, and then chilled on ice. Other digestion temperatures from 30 to 85 °C were also tested, and 65 °C was determined to be optimal for reducing rRNA secondary structure without compromising overall RNA integrity of the samples. To digest the ASOs, Turbo DNase (Invitrogen, Cat:AM2238) and the buffer were added to a final concentration of 0.1 U/µl and the reaction was incubated at 37 °C for 25 min. mRNA was then purified by RNA Clean & Concentrator-5 Kit (ZYMO, Cat:R1015) as described in (Zhang *et al.*, 2012). The RNA-seq libraries were constructed by NEBNext Ultra II RNA Library Prep kit (NEB, Cat:E7775, E7335, E7500) and sequenced by Illumina NextSeq 500 system.

**Data analysis**

Adaptor sequences were trimmed and reads shorter than 15 nt were filtered out from analysis by *Cutadapt/1.4.1* (Martin, 2011). For the analysis shown in Fig. 1F,the srpR reads were removed by initially mapping with *Bowtie2*/*2.3.4.3* (Langmead and Salzberg, 2012), and either total filtered reads (Fig. 1E) or the remaining reads after srpR removal (Fig. 1F) were mapped to *C. elegans* genome (WBcel235) by *Star/2.5.3* with default parameters (Dobin *et al.*, 2013)*.* Mapping data was sorted and processed by *Samtools/1.9* (Li *et al.*, 2009). Gene counting was performed using *featureCounts* (Liao *et al.*, 2014).

## Reagents

The ASO sequences in this research are available upon request.

## References

[R1] Arribere JA, Cenik ES, Jain N, Hess GT, Lee CH, Bassik MC, Fire AZ (2016). Translation readthrough mitigation.. Nature.

[R2] Barucci G, Cornes E, Singh M, Li B, Ugolini M, Samolygo A, Didier C, Dingli F, Loew D, Quarato P, Cecere G (2020). Small-RNA-mediated transgenerational silencing of histone genes impairs fertility in piRNA mutants.. Nat Cell Biol.

[R3] Dobin A, Davis CA, Schlesinger F, Drenkow J, Zaleski C, Jha S, Batut P, Chaisson M, Gingeras TR (2012). STAR: ultrafast universal RNA-seq aligner.. Bioinformatics.

[R4] Ellis RE, Sulston JE, Coulson AR (1986). The rDNA of C. elegans: sequence and structure.. Nucleic Acids Res.

[R5] Langmead B, Salzberg SL (2012). Fast gapped-read alignment with Bowtie 2.. Nat Methods.

[R6] Li H, Handsaker B, Wysoker A, Fennell T, Ruan J, Homer N, Marth G, Abecasis G, Durbin R, 1000 Genome Project Data Processing Subgroup. (2009). The Sequence Alignment/Map format and SAMtools.. Bioinformatics.

[R7] Liao Y, Smyth GK, Shi W (2013). featureCounts: an efficient general purpose program for assigning sequence reads to genomic features.. Bioinformatics.

[R8] Martin, M. 2011. Cutadapt removes adapter sequences from high-throughput sequencing reads. EMBnet.journal 17: 10-12.

[R9] McJunkin K, Ambros V (2017). A microRNA family exerts maternal control on sex determination in *C. elegans*.. Genes Dev.

[R10] Morgulis A, Coulouris G, Raytselis Y, Madden TL, Agarwala R, Schäffer AA (2008). Database indexing for production MegaBLAST searches.. Bioinformatics.

[R11] Mortazavi A, Williams BA, McCue K, Schaeffer L, Wold B (2008). Mapping and quantifying mammalian transcriptomes by RNA-Seq.. Nat Methods.

[R12] Ozsolak F, Milos PM (2010). RNA sequencing: advances, challenges and opportunities.. Nat Rev Genet.

[R13] Regalia M, Rosenblad MA, Samuelsson T (2002). Prediction of signal recognition particle RNA genes.. Nucleic Acids Res.

[R14] Robinson JT, Thorvaldsdóttir H, Winckler W, Guttman M, Lander ES, Getz G, Mesirov JP (2011). Integrative genomics viewer.. Nat Biotechnol.

[R15] Wang Z, Gerstein M, Snyder M (2009). RNA-Seq: a revolutionary tool for transcriptomics.. Nat Rev Genet.

[R16] Wu L, Fan J, Belasco JG (2006). MicroRNAs direct rapid deadenylation of mRNA.. Proc Natl Acad Sci U S A.

[R17] Zhang Z, Theurkauf WE, Weng Z, Zamore PD (2012). Strand-specific libraries for high throughput RNA sequencing (RNA-Seq) prepared without poly(A) selection.. Silence.

[R18] Zhao W, He X, Hoadley KA, Parker JS, Hayes DN, Perou CM (2014). Comparison of RNA-Seq by poly (A) capture, ribosomal RNA depletion, and DNA microarray for expression profiling.. BMC Genomics.

